# Synergistic Heterostructure Catalyst for Enhanced CO_2_‐to‐C2 Conversion and High‐Performance Aqueous Zn‐CO_2_ Batteries

**DOI:** 10.1002/smsc.202500434

**Published:** 2025-11-06

**Authors:** Muhammad Kashif Aslam, Iftikhar Hussain, Sidra Hameed, Liang Wang, Muhammad Ehtasham ul Haq, Ali H. Al‐Marzouqi, Maowen Xu

**Affiliations:** ^1^ Department of Chemical and Petroleum Engineering College of Engineering UAE University Al Ain 15551 Abu Dhabi United Arab Emirates; ^2^ Department of Mechanical Engineering City University of Hong Kong 83 Tat Chee Avenue Kowloon Hong Kong; ^3^ School of 210094 Engineering Nanjing University of Science and Technology Nanjing 400715 P. R. China; ^4^ Chongqing Key Laboratory of Battery Materials and Technologies School of Materials & Energy Southwest University Chongqing 400715 P. R. China

**Keywords:** C2 product, CO_2_ electroreduction, flow cell, heterostructutre, Zn‐CO_2_ battery

## Abstract

This study investigates the synergistic interaction of CuO and SnO_2_ in a heterostructure catalyst (CuO@SnO_2_) for the conversion of C1 carbon dioxide (CO_2_) reduction products to C2 products and its application in high‐performance aqueous Zn‐CO_2_ batteries. This synergistic combination enhances the Faradaic efficiency (FE) for ethanol production from 12.5% to 41.8%, shifting the selectivity from C1 to C2 products. The flow‐type aqueous Zn‐CO_2_ battery exhibits an ultrahigh power density of 6.5 mW cm^−2^, demonstrates a high discharge voltage of 0.9 V, and maintains stable operation over 140 cycles, underscoring the catalyst's exceptional reversibility and durability. During battery discharge, the system achieves a FE of 36.86% for ethanol production. These results highlight the pivotal role of the CuO@SnO_2_ synergy in optimizing CO_2_ conversion efficiency while generating electrical energy. The findings advance the development of dual‐function energy storage systems that integrate renewable electricity generation with sustainable CO_2_ utilization, paving the way for industrial‐scale applications.

## Graphical abstract

This study explores the CuO@SnO_2_ heterostructure catalyst for CO_2_ reduction, focusing on C1 to C2 product conversion and ethanol selectivity. It highlights enhanced Faradaic efficiency, catalyst synergy, and application in aqueous Zn‐CO_2_ batteries. Key aspects include improved power density, voltage stability, cycling durability, dual‐function energy storage, sustainable CO_2_ utilization, and prospects for industrial‐scale integration.

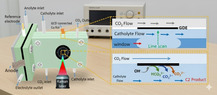

## 1 Introduction

The transformation of CO_2_ into value‐added products using renewable electricity is a potential approach to offset CO_2_ emissions and utilize renewable energy. Metal‐carbon dioxide (M‐CO_2_) batteries is an innovative technology integrating energy production with CO_2_ fixation/utilization and it have garnered significant interest for their dual ability to address environmental and energy challenges.^[^ ^1^, ^2^, ^3^, ^4^ ^]^ Among these systems, Li‐CO_2_ and Na‐CO_2_ batteries dominate current research due to their high energy density and elevated discharge voltage plateau, driving rapid advancements in the field.^[^ ^5^, ^6^, ^7^, ^8^, ^9^ ^]^ M‐CO_2_ batteries are evolving into two key applications, first one is the energy production. Li/Na‐CO_2_ systems, with their high energy output, primarily serve energy production purposes and second one is the CO_2_ fixation: Zn/Al‐CO_2_ batteries leverage their anode reactivity for chemical production, such as synthesizing value‐added products from CO_2_.^[^ ^10^, ^11^, ^12^ ^]^ The functionality of M‐CO_2_ batteries varies significantly based on the anode's electrochemical activity. Li/Na‐CO_2_ systems are primarily employed for energy production due to their high energy density, but this system face challenges like safety risks under harsh conditions and the formation of low‐value carbonates (e.g., Li_2_CO_3_/Na_2_CO_3_), which can poison and deactivate cathodes.^[^ ^13^, ^14^, ^15^ ^]^ Zn/Al‐CO_2_ systems enable chemical production (e.g., CO, HCOOH, CH_4_, CH_3_CH_2_OH) via selective CO_2_ reduction,^[^ ^16^, ^17^, ^18^ ^]^ leveraging their lower anode reactivity and simpler reaction pathways.^[^ ^19^ ^]^ Research on K/Mg‐CO_2_ batteries remains in very early stages.^[^ ^20^ ^]^ Among emerging systems, aqueous Zn‐CO_2_ batteries stand out due to their product diversity, room‐temperature operation, low Zn cost, resource abundance, and industrial scalability, positioning them as promising candidates for sustainable CO_2_ utilization.^[^ ^21^ ^]^ Aqueous Zn‐CO_2_ batteries also act as a versatile and sustainable platform for tunable CO_2_ electrochemistry, enabling the controlled synthesis of fuels, high‐value chemicals (e.g., HCOOH, CO, C_2_H_4_, and alcohols), and electricity via proton‐coupled electron transfer mechanisms. Crucially, liquid and gaseous byproducts formed during discharge are efficiently captured within the electrolyte or isolated in gas‐collection systems. This containment prevents cathode surface deposition, mitigating performance degradation and enhancing long‐term stability.

The product selectivity during CO_2_ER mainly depends on the structural evolution. Such as, the copper (Cu)‐based electrocatalysts are considered promising for the CO_2_ER due to the optimal adsorption energy of the *CO intermediate on Cu. The C—C coupling reaction involved in CO_2_ER, which produces various C_2_ products, is highly dependent on the adsorption energy of the *CO intermediate.^[^ ^22^ ^]^ In addition to C_2_ products, C_1_ products can also form on Cu‐based electrocatalysts.^[^ ^23^ ^]^ Therefore, the rational tuning of Cu‐based electrocatalysts through various modification strategies to modulate the adsorption energy of the *CO intermediate is highly favorable for enhancing the selectivity of C_2_ products over C_1_ products. Recently, different approaches have been employed for the structural modification of Cu‐based electrocatalysts to enhance the selectivity of C_2_ products.^[^ ^24^ ^]^ These approaches include the design of high‐index facet Cu nanocrystals,^[^ ^25^ ^]^ tuning surface morphology,^[^ ^26^ ^]^ and utilizing oxide‐derived copper.^[^ ^27^ ^]^ Besides this, modulating *CO adsorption energy by forming heterostructures or composites of Cu with other electrocatalysts is a common strategy for increasing C_2_ selectivity. Commonly utilized heterostructures for CO_2_ER to C_2_ products include CuAu^[^ ^28^ ^]^ and CuAg .^[^ ^29^ ^]^ However, the high cost and low abundance of noble materials hinder their application on an industrial scale. Cu‐Sn‐based electrocatalysts are considered favorable for the production of CO and HCOO^−^ due to their limited tendency toward HER, low cost, abundance, and low toxicity.^[^ ^30^, ^31^ ^]^ For example, single Sn‐atom doped Cu shows a preferential ability to form CO,^[^ ^32^ ^]^ whereas a core/shell structure of CuSn alloy/Cu‐doped SnO demonstrates remarkable efficiency toward HCOO^−^ production.^[^ ^33^ ^]^ A core/shell structure of Cu@SnO_2_ showed high activity for CO production. However, when the thickness of the SnO_2_ shell is increased, HCOO^−^ production has been observed. The thickness of SnO_2_ in Cu@SnO_2_ controls the selectivity of CO/HCOO^−^. Whereas the Cu^0^@Cu_6_Sn_5_/Sn^0^/SnO_x_ structure results in the formation of HCOO^−^. A thick SnO_2_ shell in Cu^0^@Cu_6_Sn_5_/Sn^0^/SnO_x_ acts as a barrier to H* adsorption, improving the selectivity toward HCOO^−^.^[^ ^34^ ^]^ The CO formation on Cu@SnO_2_ is due to the weak binding energy of *CO on the Sn sites. A Sn‐doped Cu bimetallic catalyst is suitable for CO production because * CO is weakened on its surface.^[^ ^35^ ^]^

The product selectivity during CO_2_ER highly depends on the structural evolution of CuO and SnO_2_. The evolved structure of the Cu‐Sn system is highly dependent on the primary structure and components of Cu‐Sn systems.^[^ ^36^ ^]^ Most research is focused on the influence of the oxidation state of Sn and binding energy optimization for improving C1 selectivity. However, the area focusing on the design of electrocatalysts while considering the synergistic interaction between Cu and Sn is not well reported. Therefore, in situ electrochemical techniques provide a unique pathway for analyzing the synergistic interaction of the Cu‐Sn system by observing the evolved structure/phases during CO_2_ER. This could further help in understanding product selectivity during CO_2_ER. The purpose of this research is to develop a CuO@SnO_2_ heterostructure system for C1 and C2 products and observe the effect of evolved phases during CO_2_ER on product selectivity. Herein, a facile oxidation method was used for the synthesis of CuO@SnO_2_ heterostructure. The evolved phase of CuO/SnO_2_ during CO_2_ER was observed using in situ techniques at different time intervals.

## 2 Results and Disuccsion

### 2.1 CO_2_ER Performance

**Figure 1:**
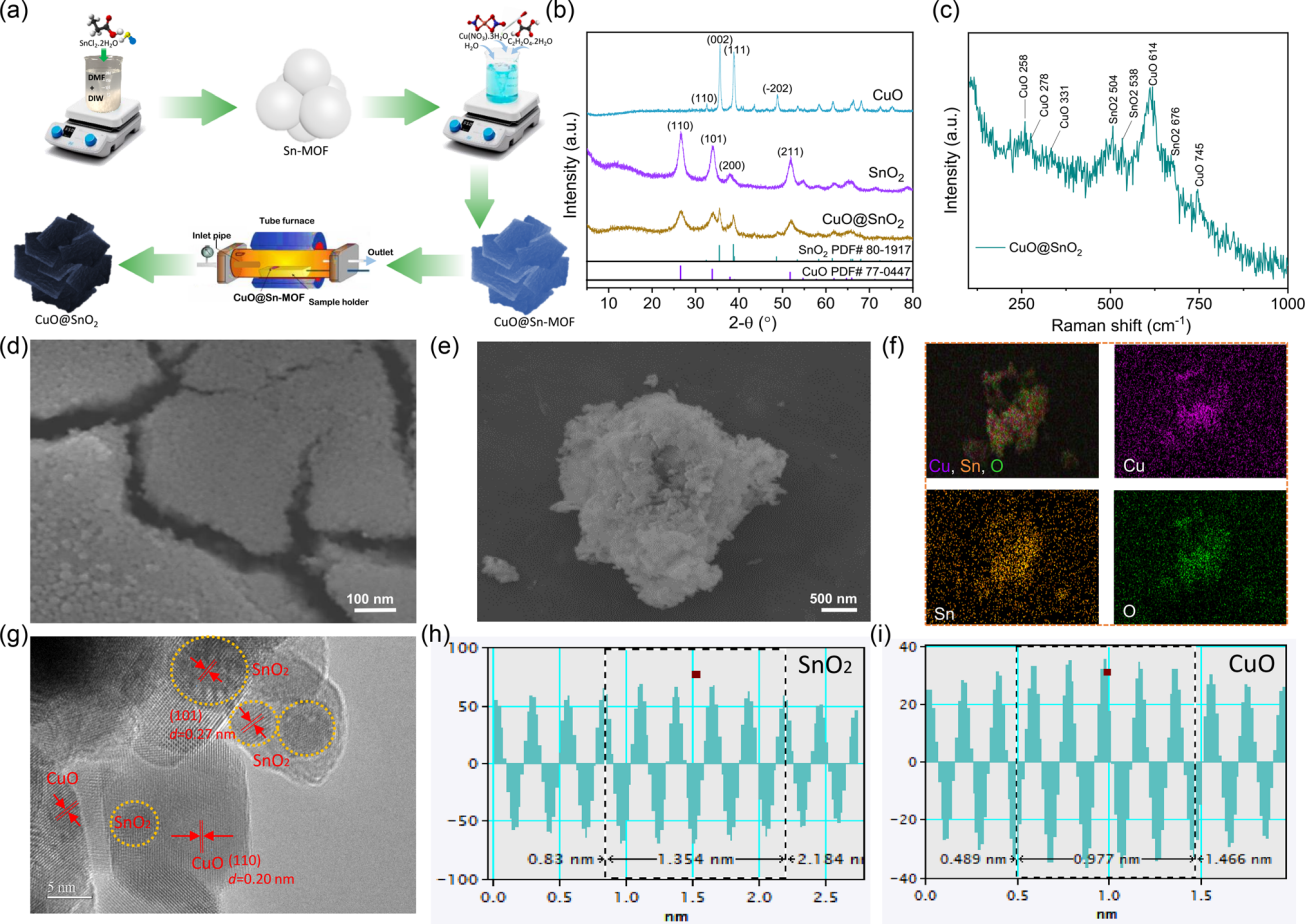
a) Synthesis scheme of CuO@SnO_2_ heterostructure, b) XRD patterns of all as synthesized materials, c) Raman spectra of CuO@SnO_2_, d,e) FESEM images of SnO_2_ and CuO@SnO_2_, respectively, f) EDS colour mapping of CuO@SnO_2_, g) high‐resolution TEM of CuO@SnO_2_, and h,i) line scanning profile for *d*‐spacing of CuO and SnO_2_.

**Figure 2:**
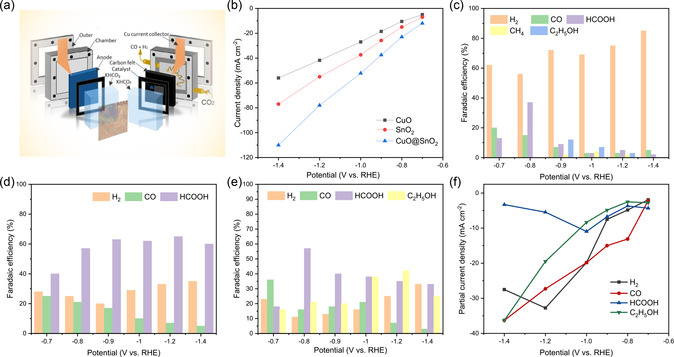
a) Configuration of flow‐cell, b) steady‐state current densities profiles of all catalysts, c) Fadaic efficiency of CO_2_ER product on CuO catalyst, d) SnO_2_ catalyst, e) CuO@SnO_2_ catalyst, and f) partial current densities displayed at different potentials.

The CuO@SnO_2_ heterostructure was obtained using calcination of single‐phase oxalate in Ar atmosphere and compared with CuO and SnO_2_ (**Figure** **1**a). The formation of heteroscuture was confirms form the X‐ray diffraction (XRD) pattern of CuO and SnO_2_ as shown in Figure 1b. Furthermore, the heterostructure was also confirmed by Raman spectroscopy. Raman spectroscopy confirms the formation of a CuO@SnO_2_ heterostructure by detecting the characteristic vibrational modes of both CuO (peaks at ≈278, 331, and 614 cm^−1^) and SnO_2_ (peaks at ≈504, 538, 649 676, and 745 cm^−1^) in the composite spectrum, ensuring their coexistence. Interactions at the interface are evidenced by subtle peak shifts from the original ones and broadening,^[^ ^37^ ^]^ indicative of interfacial strain or charge transfer, while the absence of secondary‐phase peaks confirms structural integrity. The technique's surface sensitivity may highlight SnO_2_‐dominated signals in a spherical configurations, and defect‐related features (e.g., oxygen vacancies) further validate interfacial interactions. This analysis distinguishes the heterostructure from physical mixtures or new chemical phases, confirming successful synthesis (Figure 1c). The nanosized (5 to 15 nm) sphere‐shaped Sn‐MOF particles were first synthesized (Figure 1d), and then the heterostructured CuO@SnO_2_ was prepared. The morphoglical structure of CuO@SnO_2_ structure resemble the flakes as revealed by field emission scanning electron microscopy (FESEM) (Figure 1e). The energy dispersive X‐ray spectroscopy (EDS) mapping confirms the presence of Cu, Sn and O as shown in Figure 1f. The FESEM images and EDS mapping of CuO and SnO_2_ individuals also given in supporting information (Figure S1–3, Supporting Information). High‐resolution transmission electron microscopy (HRTEM) reveals the (^−^111) and (110) planes of CuO and SnO_2_ in the CuO@SnO_2_ heterostructure with interlayer spacing of 0.20 nm and 0.27 nm, respectively (Figure 1g,h,i). The synthesis of heterostructure CuO@SnO_2_ is further confirmed by the prominent interfaces between the CuO and SnO_2_ as shown in Figure 1 g and S4,5, Supporting Information. The CO_2_ electroreduction (CO_2_ER) selectivity and activity of the synthesized catalysts were evaluated in a flow‐type cell separated by a Nafion 117 membrane, using CO_2_‐saturated 0.5 M KHCO_3_ electrolyte (**Figure** **2**a).

To establish a robust correlation between in situ structural evolution and catalytic performance, all catalysts were initially subjected to linear sweep voltammetry (LSV) until stable LSV profiles were attained. Notably, the stabilized LSV curves (Figure S6, Supporting Information) indicate that the CuO@SnO_2_ heterostructure undergoes in situ structural and phase reconstruction during CO_2_ER. The CO_2_ER performance was systematically assessed through chronopotentiometry (i–t) testing as well, with polarization curves generated using steady‐state current density measurements (Figure 2b). Consistent with these results, steady‐state LSV supported that the CuO@SnO_2_ heterostructure exhibits superior CO_2_ER activity compared to individual CuO and SnO_2_ catalysts. Gas chromatography and ^1^H nuclear magnetic resonance (^1^H NMR) spectroscopy were utilized to quantify CO_2_ER products and assess catalyst selectivity. For the CuO catalyst, H_2_ dominated as the primary product, exhibiting a FE exceeding 85% at −1.4 V versus reversible hydrogen electrode (RHE). Additionally, HCOO^−^ was detected at a lower overpotential (−0.8 V versus RHE), achieving an FE of 38% for formic acid (Figure 2c). The FE for HCOO^−^ declines sharply at potentials more negative than −0.8 V versus RHE, while ethanol emerges as a new product, attaining an FE of 12.53% for CH_3_CH_2_OH at −0.9 V versus RHE (Figure 2c). For SnO_2_ catalyst, the hydrogen evolution reaction (HER) is significantly suppressed, with C1 products (CO and HCOOH) dominating across the tested potentials. Notably, SnO_2_ achieves a maximum FE of 65% for HCOOH at −1.2 V versus RHE (Figure 2d). In contrast to pure SnO_2_, the CuO@SnO_2_ heterostructure generates four products: CH_3_CH_2_OH, HCOOH, H_2_, and CO (Figure 2e). At −0.7 V versus RHE, CO is the primary product, reaching a peak FE of 35% within the studied potential range. As the overpotential increases (−0.75 to −1.0 V vs. RHE), HCOOH becomes dominant, achieving a maximum FE of 57.5% with a partial current density (J_HCOOH_) of 11.5 mA cm^−2^ at −0.8 V versus RHE. At higher overpotentials, CH_3_CH_2_OH emerges as the major product (validated by ^1^H NMR in Figure S7, Supporting Information), with its FE peaking at 41.80% and a partial current density of −20 mA cm^−2^ at −1.2 V versus RHE (Figure 2f). This ethanol production rate is ≈3.37 times higher than that of CuO‐derived catalyst. Importantly, the CuO@SnO_2_ heterostructure at −1.2 V versus RHE demonstrates competitive ethanol selectivity compared to other Cu‐based electrocatalysts in prior studies (Table S1, Supporting Information).

Notably, CO is a critical intermediate in ethanol (CH_3_CH_2_OH) formation during CO_2_ER, exhibits a declining FE with increasing overpotential. This trend suggests that enhanced CO intermediate generation at higher overpotentials facilitates C—C coupling, enabling ethanol production. The results imply that the pristine CuO@SnO_2_ heterostructure undergoes structural and phase reconstruction under varying potentials, likely governing the shift in dominant CO_2_ER products. Specifically, product selectivity for CuO@SnO_2_ at −0.8 V versus RHE diverges sharply from trends observed at −1.2 V versus RHE (where ethanol dominates). This behavior aligns with the catalyst's potential‐dependent structural evolution: the phase restructured at −1.2 V versus RHE transitions to a stabilized CuO@SnO_2_ configuration when the potential is adjusted to −0.8 V versus RHE. Consequently, selectivity at −0.8 V versus RHE over CuO@SnO_2_‐1.2 V closely resembles that of CuO@SnO_2_‐0.8 V. These findings reaffirm that CO_2_ER product selectivity is inherently structure‐dependent, with the catalyst's architecture being dynamically modulated by the applied potential.

**Figure 3:**
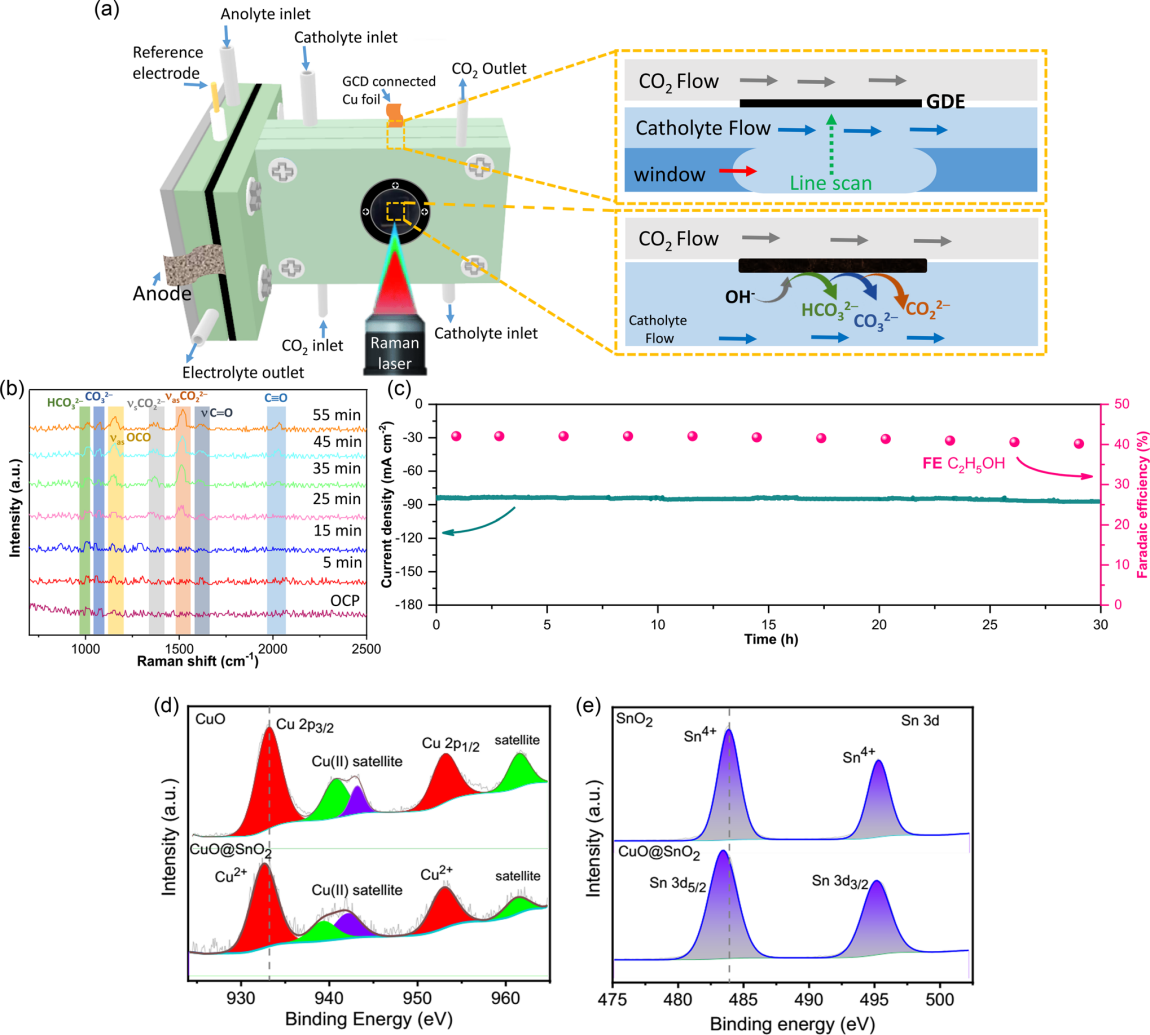
a) Configuration depiction of cell used for in situ Raman spectroscopy, b) in situ Raman spectra's during CO_2_ER at different time intervals, c) CuO@SnO_2_ catalytic stability and corresponding FE's at −1.2 V, and d,e) High‐resolution XPS spectrums of Cu and Sn of individuals (CuO and SnO_2_) and in heterostructured CuO@SnO_2_.

The CuO@SnO_2_ heterostructure facilitates C—C coupling as well and product selectivity modulation, as evidenced by X‐Ray photoelectron spectroscopy (XPS) analysis. In the heterostructure, Cu and Sn binding energies exhibit negative shifts relative to standalone CuO and SnO_2_, a phenomenon linked to interfacial electronic interactions and charge redistribution. The *p*‐n junction formed between p‐type CuO and n‐type SnO_2_ drives electron transfer from SnO_2_ to CuO, equilibrating their Fermi levels. This results in electron enrichment at Sn sites (lowering Sn^4+^ BE) and partial reduction of Cu^2+^ to Cu^+^/Cu^0^ (reducing Cu BE) (**Figure** **3**d,e). Oxygen vacancies and interfacial strain further redistribute charge, lowering effective nuclear charges. These electronic adjustments enhance CO_2_ER efficiency by optimizing *CO intermediate adsorption and enabling C—C coupling, preferentially steering ethanol formation at elevated overpotentials. The interplay of charge transfer, defects, and structural strain tailors catalytically active sites for selective product generation.

In situ Raman spectroscopy was used to investigate the reconfiguration of phases CuO@SnO_2_ during electrochemical conversion of CO_2_ as shown in Figure 3a. To investigate the distinct intermediates involved in formate and ethanol production, time‐resolved operando Raman spectroscopy was employed to track the evolution and dynamics of adsorbates on the CuO@SnO_2_ heterostructure during CO_2_ER at varying potentials. Figure 3b displays the Raman spectra of the heterostructure under CO_2_‐saturated electrolyte at −1.2 V versus RHE. Two prominent peaks at 1012 cm^−1^ (assigned to surface‐adsorbed HCO_3_^−^) and 1068 cm^−1^ (linked to CO_3_^2−^) are observed. As CO_2_ER progresses, new peaks emerge at 1370 cm^−1^ and 1526 cm^−1^, corresponding to the symmetric and asymmetric stretching vibrations of the *CO_2_^−^ intermediate, respectively. These spectral features provide direct evidence of adsorbate transformations during the reaction, correlating with the heterostructure's catalytic behavior. At 25 min, Raman spectra reveal two characteristic peaks: one at ≈1151 cm^−1^, attributed to OCO antisymmetric stretching vibrations, and another at ≈1630 cm^−1^, corresponding to the C=O stretching mode of *COOH, a critical intermediate in *CO formation. By 35 min, a distinct peak emerges at ≈2046 cm^−1^, assigned to the C≡O stretching vibration of *CO adsorbed on the Cu surface, with its intensity increasing progressively over time. These time‐resolved in situ Raman observations demonstrate that the evolving Cu@SnO_2‐__x_ heterostructure under an applied potential of −1.2 V versus RHE facilitates the generation and stabilization of *CO. This key intermediate enhances C—C coupling, thereby promoting the selective production of C_2+_ products.

To evaluate the stability of the CO_2_ER system enhanced by CuO@SnO_2_, chronoamperometric testing was performed at a fixed potential of −1.2 V versus RHE (Figure 3c). The CuO@SnO_2_‐based electrode exhibited stable operation over 30 h, retaining a high Faradaic efficiency for ethanol (FEEthanol) of 41.5%. During the 30 h test, the ethanol production rate displayed only a minor decline, and the strong linear correlation between ethanol concentration and testing duration further confirmed the system's robust stability. Notably, the CO_2_ER performance of CuO@SnO_2_ rivals that of leading reported catalysts for ethanol production, underscoring its practical relevance.

### 2.2 Aqueous Zn‐CO_2_ Battery Performance

**Figure 4:**
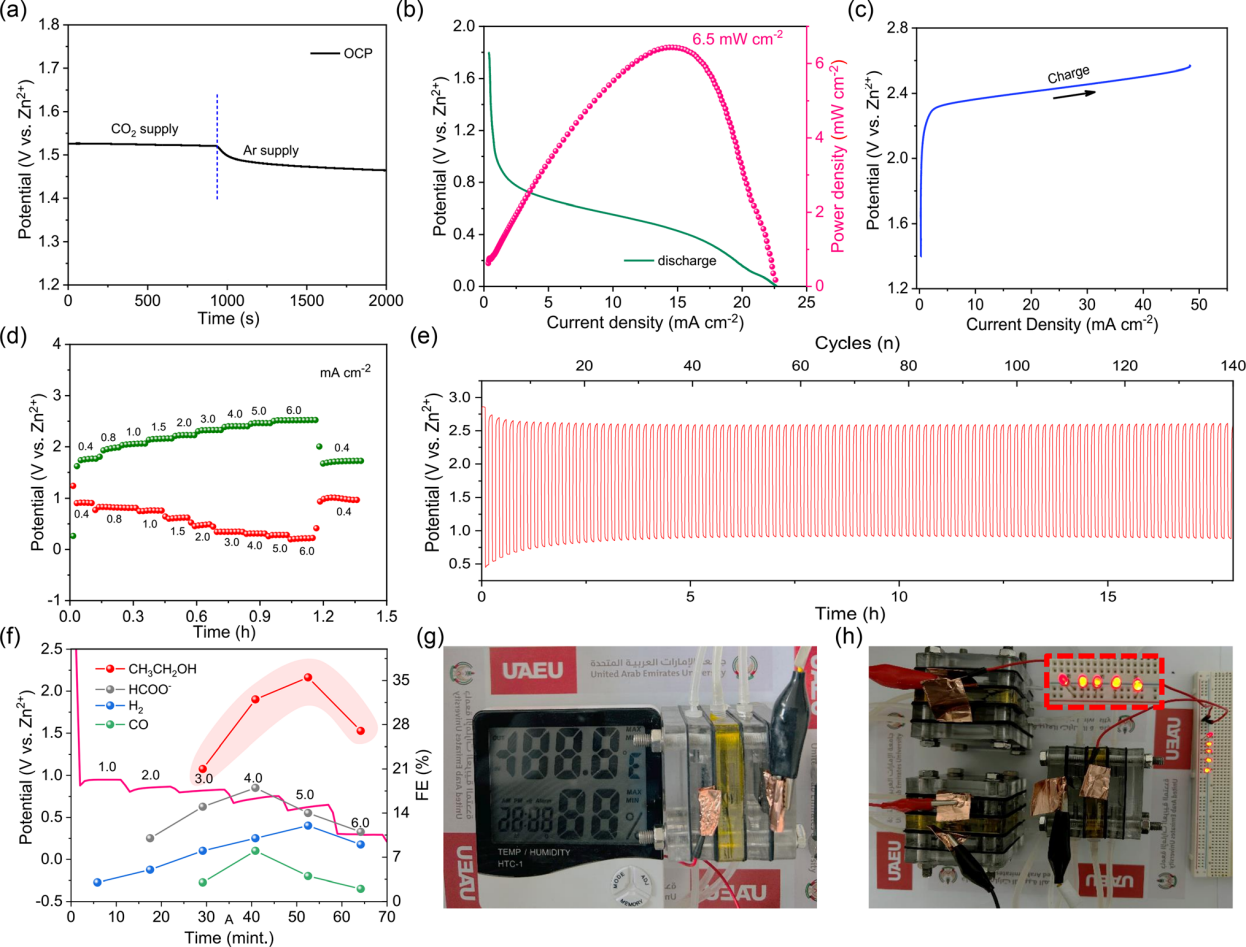
Electrochemical measurements of aqueous Zn‐CO_2_ battery. a) OCP curve of as assembled Zn‐CO_2_ battery under continuous supply of CO_2_ and Ar gas, b) polarization discharge curve and corresponding power density curve, c) polarization charge curve, d) rate capability test under different current densities, e) cycling stability test at 0.4 mA g^−1^, f) continues discharge under different current densities and corresponding FE's of CO_2_ER products during battery discharge at each current density, and g,h) visible photos of digital clock and LED's powered by aqueous Zn‐CO_2_ battery.

Building on the exceptional CO_2_ER performance of CuO@SnO_2_, a functional aqueous Zn‐CO_2_ battery was constructed using a flow‐cell configuration, illustrated in Figure 2a replacing anion exchange membrane (AEM) with BPM. This flow‐type aqueous Zn‐CO_2_ battery achieves a notable open‐circuit potential (OCP) of 1.53 V under continuous CO_2_ gas purging. Intriguingly, replacing the CO_2_ environment with Ar gas induces an abrupt voltage shift in the OCP profile, as observed in **Figure** **4**a. Additionally, the flow‐cell design delivers robust discharge performance, attaining a peak power density of 6.5 mW cm^−2^ at 15 mA cm^−2^ (Figure 4b), a marked improvement over prior studies (Table S2, Supporting Information), representing the highest reported value to date. The battery was charged to evaluate its charge voltage and voltage gap. As shown in Figure 4c, the charge voltage remains significantly below 3 V, resulting in high energy efficiency and a low voltage gap (53% and 0.82 V @0.4 mA cm^−2^, respectively), which enhances overall battery performance. Rate capability tests, critical for assessing stability across varying current densities, were conducted by cycling the battery at 0.4, 0.8, 1.0, 1.5, 2.0, 3.0, 4.0, 5.0, and 6.0 mA cm^−2^. Remarkably, the battery maintains stable and elevated discharge voltage plateaus at all tested current densities (Figure 4d), underscoring its operational reliability.

Notably, when the current density is cycled back to 0.4 mA cm^−2^ after operating at 6 mA cm^−2^, the voltage reverts to its original level, highlighting the robust stability of the CuO@SnO_2_ cathode under high‐current‐density conditions. The aqueous Zn‐CO_2_ battery achieves exceptional discharge voltages of 0.96, 0.89, 0.8, 0.7, 0.57, 0.4, 0.37, 0.36, and 0.3 V at current densities of 0.4, 0.8, 1, 1.5, 2, 3, 4, 5, and 6 mA cm^−2^, respectively. These values, particularly at elevated current densities, are rarely documented in comparable systems.

Moreover, the charging voltage critically reduces the voltage gap. During charging in the flow‐type Zn‐CO_2_ battery, the formate and ethanol produced via CO_2_ER in the discharge phase undergoes oxidation. This oxidation of discharge‐generated liquid products (CO_2_ER) confirms the system's reversibility. As oxidation progresses, the charging voltage diminishes, narrowing the voltage gap and enhancing energy efficiency. Impressively, the flow Zn‐CO_2_ battery sustains a charging voltage below 3.0 V even at 6 mA cm^−2^ (Figure 4d).

The reversibility, cycling stability, and synergistic interaction between CuO and SnO_2_ were systematically evaluated through extended charge/discharge cycling tests, as depicted in Figure 4e. The flow‐type battery maintains a discharge voltage of 0.9 V and a charging voltage of 2.57 V under operational conditions. Figure 4e further reveals that the CuO@SnO_2_ cathode enables stable performance over 140 cycles, emphasizing its exceptional reversibility, attributed to the cooperative effects of CuO and SnO_2_.

To assess practical scalability and quantify CO_2_ conversion efficiency, the Zn‐CO_2_ battery was discharged at varying current densities, with CO_2_ER products analyzed. As illustrated in Figure 4f, the battery achieves a FE for ethanol of 36.86% even at elevated current densities, underscoring its dual capability of energy generation and CO_2_‐to‐chemical conversion. Beyond Tafel analysis, the electrochemically active surface area (ECSA) of the catalysts were determined via double‐layer capacitance (*C*dl) measurements to evaluate and quantify the materials activity.^[^ ^38^ ^]^ The *C*dl value for CuO@SnO_2_ (1.68 mF cm^−2^) surpasses those of CuO (0.44 mF cm^−2^) and SnO_2_ (0.91 mF cm^−2^), indicating its significantly larger active surface area (Figure S8–10, Supporting Information). This enhanced surface area promotes efficient CO_2_ adsorption and activation, directly correlating with improved CO_2_ electroreduction (CO_2_ER) performance (Figure S8–S10, Supporting Information). To further quantify the enhanced performance of the aqueous Zn‐CO_2_ battery, we present a comparative analysis of power densities and their corresponding current densities in Figure S11, Supporting Information. Additionally, the practical viability of the assembled battery was evaluated under real‐world conditions. As demonstrated in Figure 4g,h, the Zn‐CO_2_ battery reliably powers a temperature‐humidity digital clock and illuminates five LEDs continuously for multiple hours, underscoring its potential for low‐power electronic applications.

## 3 Conclusion

This study demonstrates the successful integration of a CuO@SnO_2_ heterostructure cathode into an aqueous Zn‐CO_2_ battery, achieving dual functionality of efficient CO_2_ conversion to multicarbon products and high‐performance energy storage. The synergistic interaction between CuO and SnO_2_ enables a remarkable shift in selectivity from C1 to C2 products, elevating the FE for ethanol production from 12.5% to 41.8%. The flow‐type Zn‐CO_2_ battery exhibits exceptional performance, delivering an ultrahigh‐power density of 6.5 mW cm^−2^, a stable discharge voltage of 0.9 V, and robust cyclability over 140 cycles. During discharge, the system maintains a competitive FE of 36.86% for ethanol, underscoring the cathode's durability and electrochemical reversibility. These results not only highlight the critical role of heterostructure engineering in optimizing CO_2_ reduction pathways but also establish a scalable framework for dual‐function energy systems that simultaneously address renewable energy storage and carbon utilization. This work paves the way for industrial‐scale applications, offering a sustainable strategy to decarbonize energy technologies while producing value‐added chemicals. Future efforts will focus on refining heterostructure interfaces and scaling battery design to further enhance efficiency and practicality.

## 4 Experimental Section

4.1

4.1.1

##### Chemicals

All of the chemicals, including copper nitrate tri‐hydrate (Cu(NO_3_)_2_·3H_2_O, Sigma–Aldrich (≥99.0%), Tin chloride dihydrate (SnCl_2_·2H_2_O, Aladdin chemicals ≥98.0%), phthalic acid Sigma–Aldrich (≥99.0%), ethanedioic acid dihydrate (C_2_H_2_O_4_·2H_2_O, Aladdin chemicals (≥99.9%), ethanol (CH_3_CH_2_OH, ≥99.9%), isopropanol (C_3_H_8_O, ≥99.7%), Nafion 117 solution (Sigma Aldrich 5 wt%), DMF and NaOH (≥99.9%) were used without further purification.

##### Synthesis of Sn‐MOF

All procured chemicals were of analytical grade and used as received. To synthesize Sn‐MOF, 2.4 mM sodium hydroxide and 1.2 mM phthalic acid were dissolved in a 60 mL solution of DMF (dimethylformamide) and DI (deionized) water at a volume ratio of 1:1 to form mixture A. Concurrently, a 0.015 M solution of tin sulfate was prepared by dissolving it in 10 mL of DI water to form mixture B. Subsequently, mixture B was rapidly added to mixture A under rigorous stirring, which continued at 50 °C for 2 h. After this period, the resulting white precipitates were washed thrice with DMF before being transferred to DI water. Once the precipitates settled, the water was replaced three times. Finally, the precipitates were dried under vacuum conditions at 60 °C.

##### Synthesis of CuO@SnO_2_ Heterostructure

First, 60 mmol of Cu(NO)_3_.3H_2_O were slowly dissolved in 40 mL of deionized water and vigorously stirred for 20 mint at room temperature and then added 30 mg of as prepared Sn‐MOF precursor (labeled as solution A); and then 60 mmol of C_2_H_2_O_4_.2H_2_O were added into 40 mL of deionized water and kept it for stirring for 30 mint under 50 °C in order to make it dissolve sufficiently (labeled as solution B). Subsequently, solution B was added into the solution A slowly under the magnetic stirring and continued stirring for 60 min. After being stirred, the blue precipitates were washed with water and ethanol five times, respectively. Finally placed the obtained powder in a drying oven at 60 °C for 12 h to obtain the oxalate precursor. In tube furnace, the precursor was heated at 400 °C with a heating rate of 5 °C min^−1^ and kept at this temperature for 2 h and then cooled down to room temperature to obtain the CuO@SnO_2_. Pure CuO was prepared same method but without the addition of Sn‐MOF precursor.

##### CO_2_ER Measurements

All CO_2_ electrocatalytic reduction measurements were carried out in a three‐chamber flow cell. The KHCO_3_ (0.5 M) electrolyte was pumped continuously into the cathodic and anodic chambers at a flow rate of 5 mL per minute. An AEM separating the anodic and cathodic chambers prevented the reduced liquid products from mixing with the anolyte. Additionally, CO_2_ was purged into the third chamber, known as the gas chamber, and beside the gas diffusion electrode layer at a flow rate of 80 sccm. All electrochemical experiments were conducted using a CHI760e workstation. A titanium plate coated with iridium and a KCl‐saturated Ag/AgCl electrode served as the counter electrode and reference electrode, respectively. The working electrode was prepared from the catalyst mixed with isopropyl alcohol and DI water (3:1 ratio), along with Nafion solution, and supported on gas diffusion carbon paper. LSV curves were recorded over a potential range from 0 to −3 V at a scan rate of 5 mV per second. The ECSA was determined between −0.2 and 0.09 V. All potentials in all electrochemical experiments were converted to the RHE reference scale using the following equation.

(1) $$E_{\text{RHE}}=E_{\text{Ag/AgCl}}+0.0591\times \text{pH}+0.197\text{ V}$$

*Zn‐CO*_*2*_ *battery assembly:* Anode preparation: First, zinc metal plate was polished with sand paper and then ultrasonically cleaned with acetone and ultrapure water (50% each), then cut it into the size of 3 × 3 cm and dry it for standby. The electrolyte of the anode was 6M KOH + 0.02 M of zinc acetate to get a certain concentration of Zn ions. Preparation of cathode: 10 mg of CuO@SnO_2_ catalyst was taken with isopropanol and DI water in the ratio of 3:1 and ultrasound for 1 hr. A spray gun was used to spray ≈6 mg of the sample onto the hydrophobic carbon paper (3 × 3 cm) and dried on hot surface. The area of the sample coated on the carbon paper was 1 × 1 cm. The catholyte used in cathode chamber was CO_2_‐saturated 0.8 M KHCO_3_. In fact, we utilized a KHCO_3_ solution as a catholyte in CO_2_ER and achieved over 40% production of CH_3_CH_2_OH. This is why we employed same catholyte in the Zn‐CO_2_ battery, aiming to replicate the same electrolyte effect observed in CO_2_ER and the battery. 3) Battery assembly: The anode and cathode electrodes of Zn‐CO_2_ battery were separated with BPM membrane and both chambers were filled with anolyte and catholyte with an extra gas chamber on cathode side for gas flow. The charge–discharge polarization curves were collected at the scan rate of 10 mV s^−1^ under continues supply of CO_2_ gas.

##### Characterizations

The structural morphology and composition of the catalyst were analyzed by scanning electron microscopy (SEM) and HRTEM, (JEM‐2100 F, 200 kV), respectively. XRD, (Bruker, D8 Advance A25, Co target, Kα 1 = 1.78897 Å, 0.02 mm thick Fe filter) was used to check purity and crystallinity of as prepared catalytic material. XPS, (PHI 5000 VersaProbe) was also performed to confirm the oxidation states of the atoms.

## Supporting Information

Supporting Information is available from the Wiley Online Library or from the author.

## Conflict of Interest

The authors declare no conflict of interest.

## Supporting information

Supplementary Material

## Data Availability

The data that support the findings of this study are available from the corresponding author upon reasonable request.
