# Supplementary material for: Synergistic Heterostructure Catalyst for Enhanced CO2‐to‐C2 Conversion and High‐Performance Aqueous Zn‐CO2 Batteries
Source: Small Sci. 2025 Nov 6;5(12):e202500434. doi: 10.1002/smsc.202500434 (PMC12697881; doi:10.1002/smsc.202500434)
Supplement: Supplementary file 1 — Supplementary Material [file SMSC-5-e202500434-s001.pdf]

Supporting information

Synergistic Heterostructure Catalyst for Enhanced CO_2-to-C2 Conversion and High-Performance Aqueous Zn-CO_2 Batteries

Muhammad Kashif Aslam [a*], Iftikhar Hussain [c], Sidra Hameed [d], Liang Wang [b], Muhammad Ehtasham-ul-Haq [d], Ali H. Al-Marzouqi [*a], Maowen Xu [*b]

[a] Department of Chemical and Petroleum Engineering, College of Engineering, UAE University, Al Ain 15551, Abu Dhabi, United Arab Emirates.

[b] Chongqing Key Laboratory of Battery Materials and Technologies, School of Materials & Energy, Southwest University, Chongqing 400715, P.R. China.

[c] Department of Mechanical Engineering, City University of Hong Kong, 83 Tat Chee Avenue, Kowloon, Hong Kong

[d] School of 210094 Engineering, Nanjing University of Science and Technology, Nanjing, 400715, P.R. China.

Corresponding Authors: M. K. Aslam (aslam_kashif@outlook.com) Ali Al-Marzouqi (hassana@uaeu.ac.ae) and Maowen Xu (xumaowen@swu.edu.cn)

Supporting Figures and Tables

Figure S1. FESEM images of CuO (a), SnO_2 (b) and CuO@SnO_2 (c).

Figure S2. EDS colour mapping of CuO.

Figure S3. EDS colour mapping of SnO_2.

Figure S4. HRTEM of CuO (a), FFT image of CuO HRTEM (b), inverse FFT (c) and line mapping of inverse FFT for *d*-spacing (d).

Figure S5. HRTEM of SnO_2 (a), FFT image of SnO_2 HRTEM (b), inverse FFT (c) and line mapping of inverse FFT for *d*-spacing (d).

Figure S6. LSV curves of as-synthesized catalysts.

Figure S7. ^{1}H NMR spectrum of CO^2ER liquid product.

Figure S8. CV curves of CuO@SnO_2 at different scan rates (a) and double layer capacitance (b).

Figure S9. CV curves of SnO_2 at different scan rates (a) and double layer capacitance (b).

Figure S10. CV curves of CuO at different scan rates (a) and double layer capacitance (b).

Figure S11. Comparison of the power densities reported in previous studies with that of this work.

Table S1. Comparison of the catalytic CO_2 electroreduction (CO_2ER) performance of CuO@SnO_2 with previously reported selective catalysts for ethanol production.

Table S2. Detailed comparison of aqueous Zn-CO_2 battery with previously reported works.

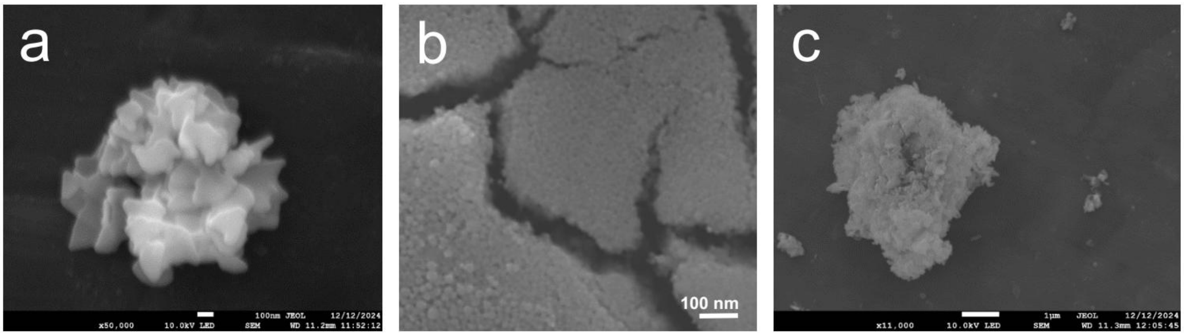

Figure S1. FESEM images of CuO (a), SnO_2 (b) and CuO@SnO_2 (c).

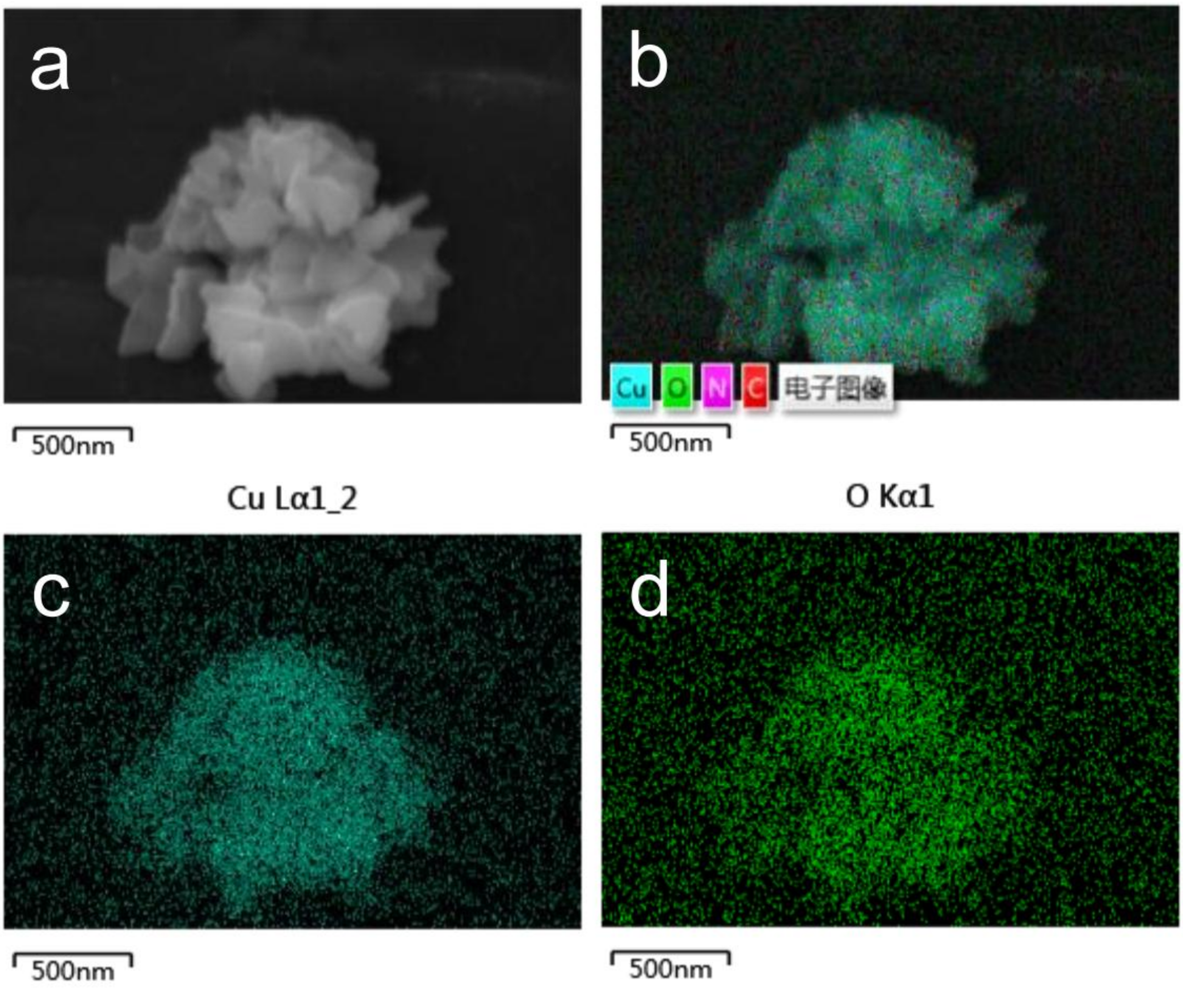

Figure S2. EDS colour mapping of CuO.

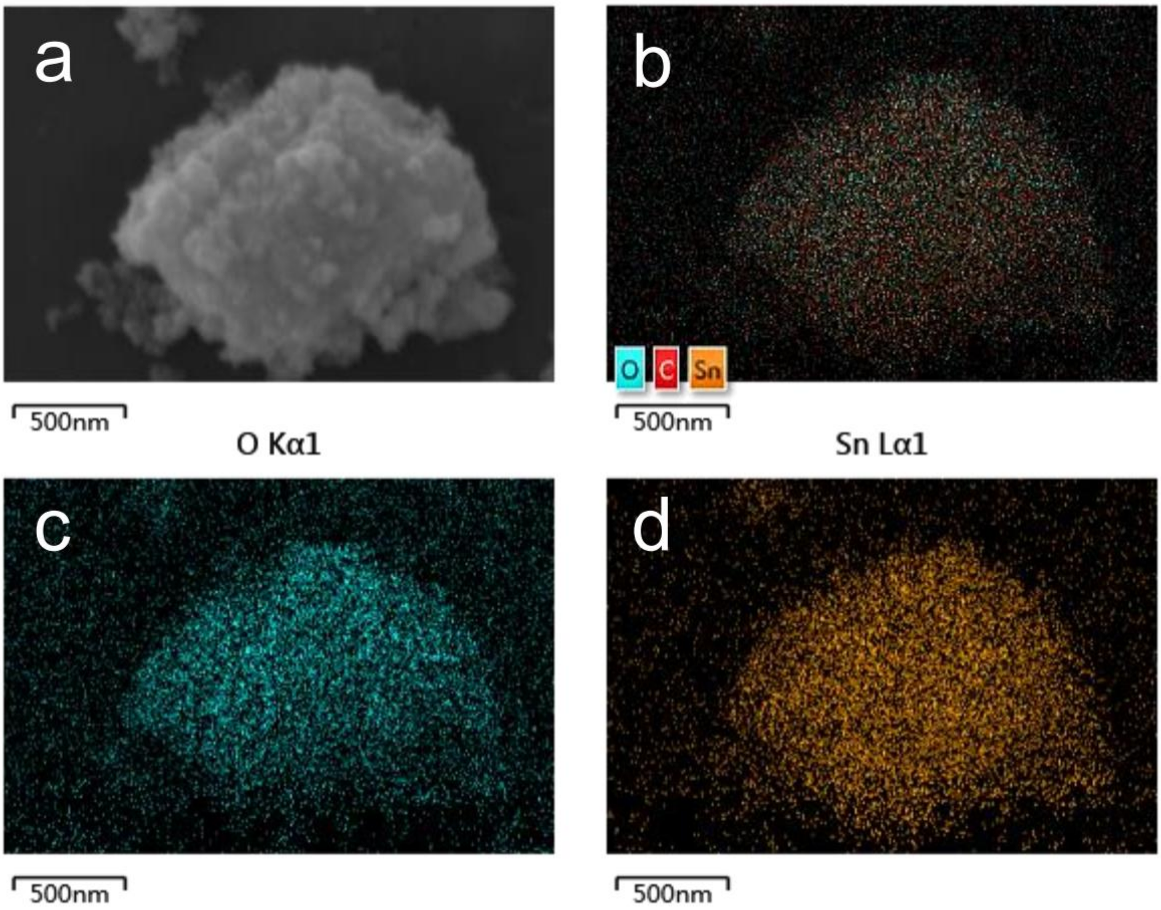

Figure S3. EDS colour mapping of SnO_2.

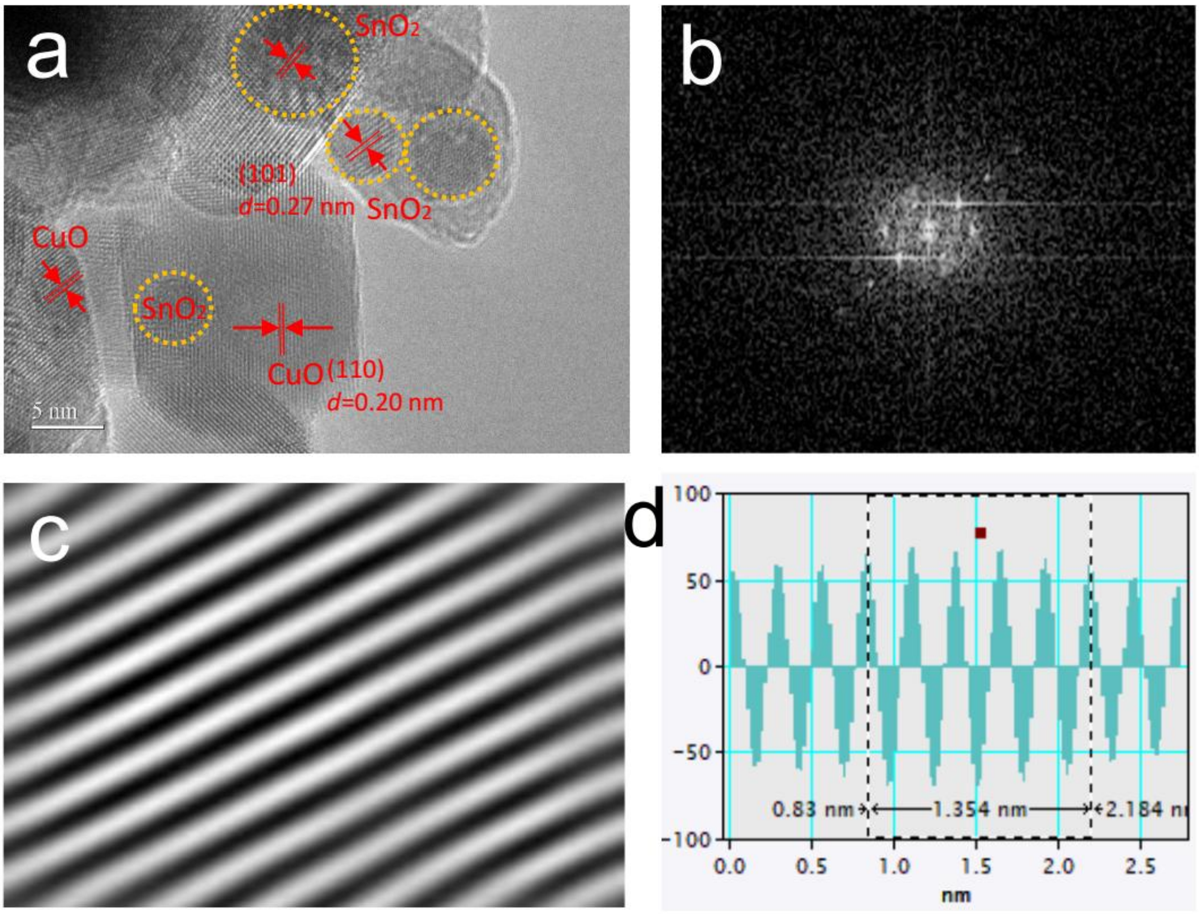

Figure S4. HRTEM of CuO (a), FFT image of CuO HRTEM (b), inverse FFT (c) and line mapping of inverse FFT for *d*-spacing (d).

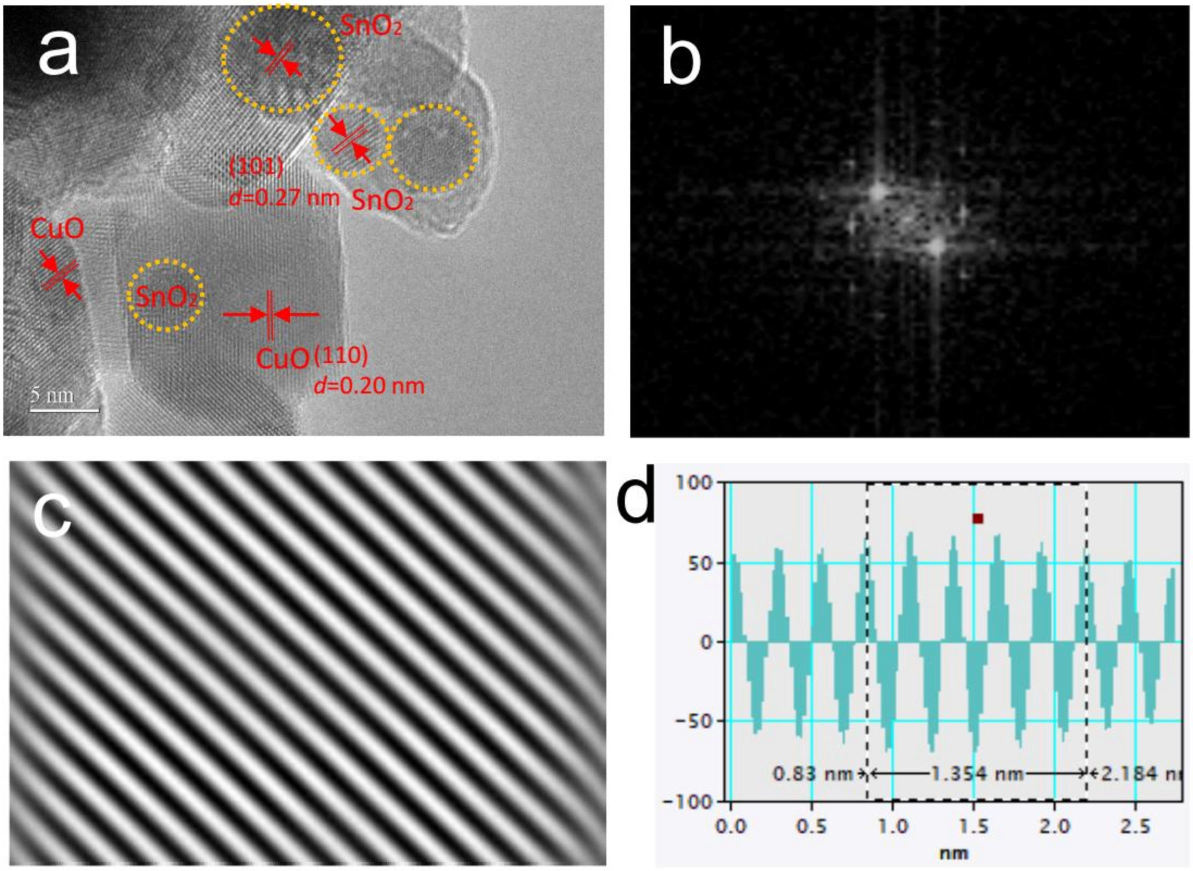

Figure S5. HRTEM of SnO_2 (a), FFT image of SnO_2 HRTEM (b), inverse FFT (c) and line mapping of inverse FFT for *d*-spacing (d).

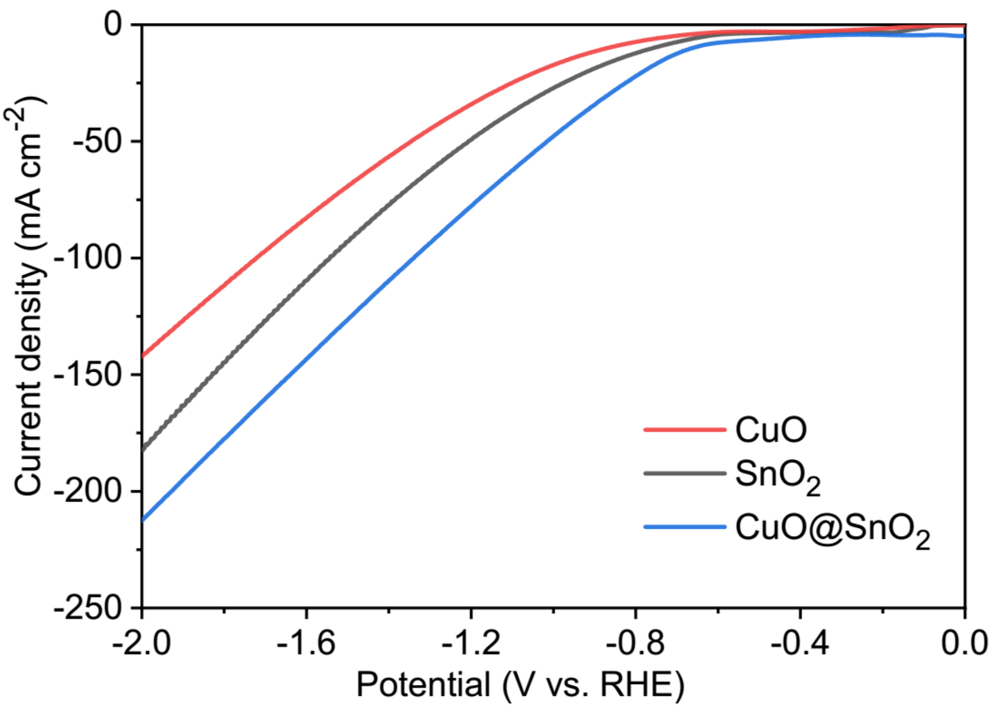

Figure S6. LSV curves of as-synthesized catalysts.

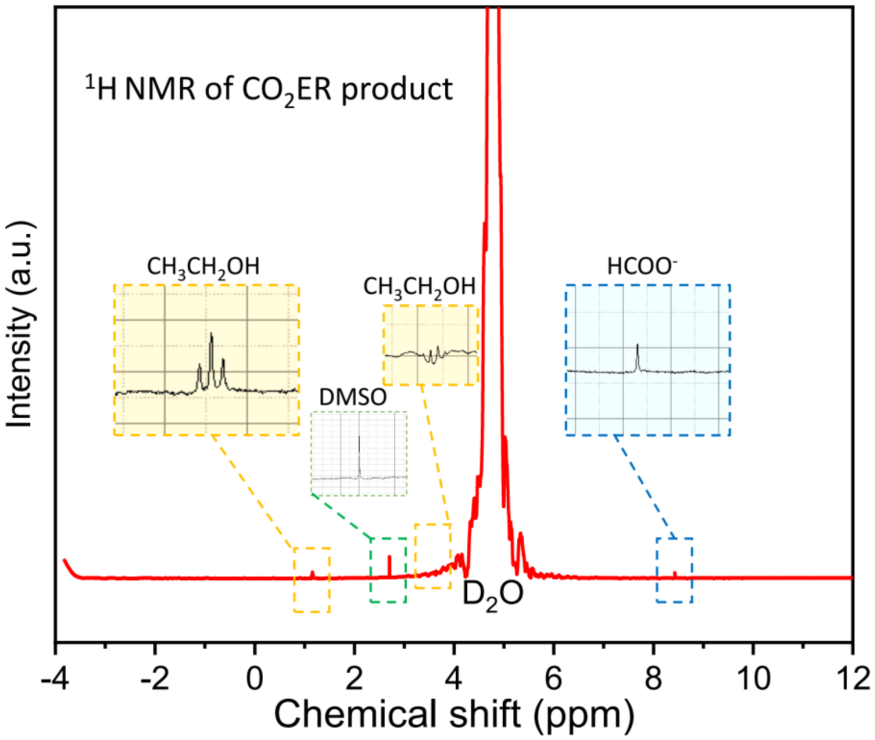

Figure S7. ^{1}H NMR spectrum of CO[2]ER liquid product.

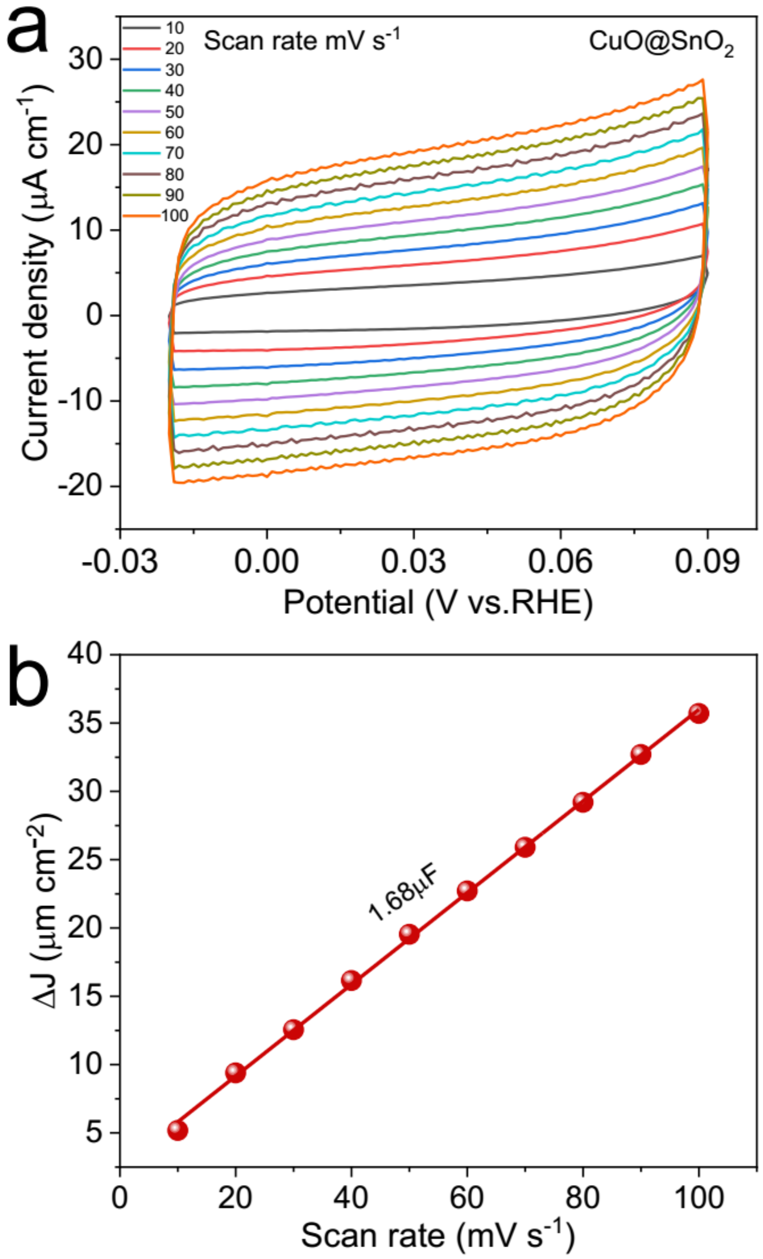

Figure S8. CV curves of $CuO@SnO_2$ at different scan rates (a) and double layer capacitance (b).

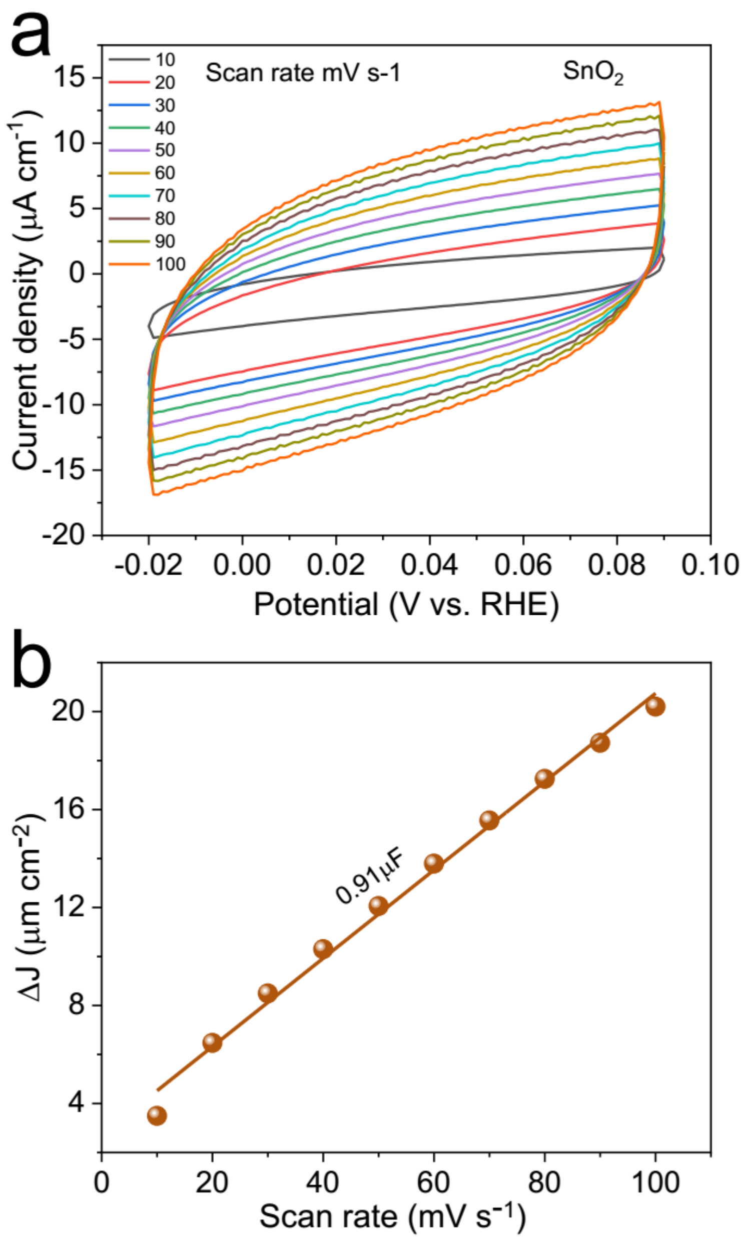

Figure S9. CV curves of SnO_2 at different scan rates (a) and double layer capacitance (b).

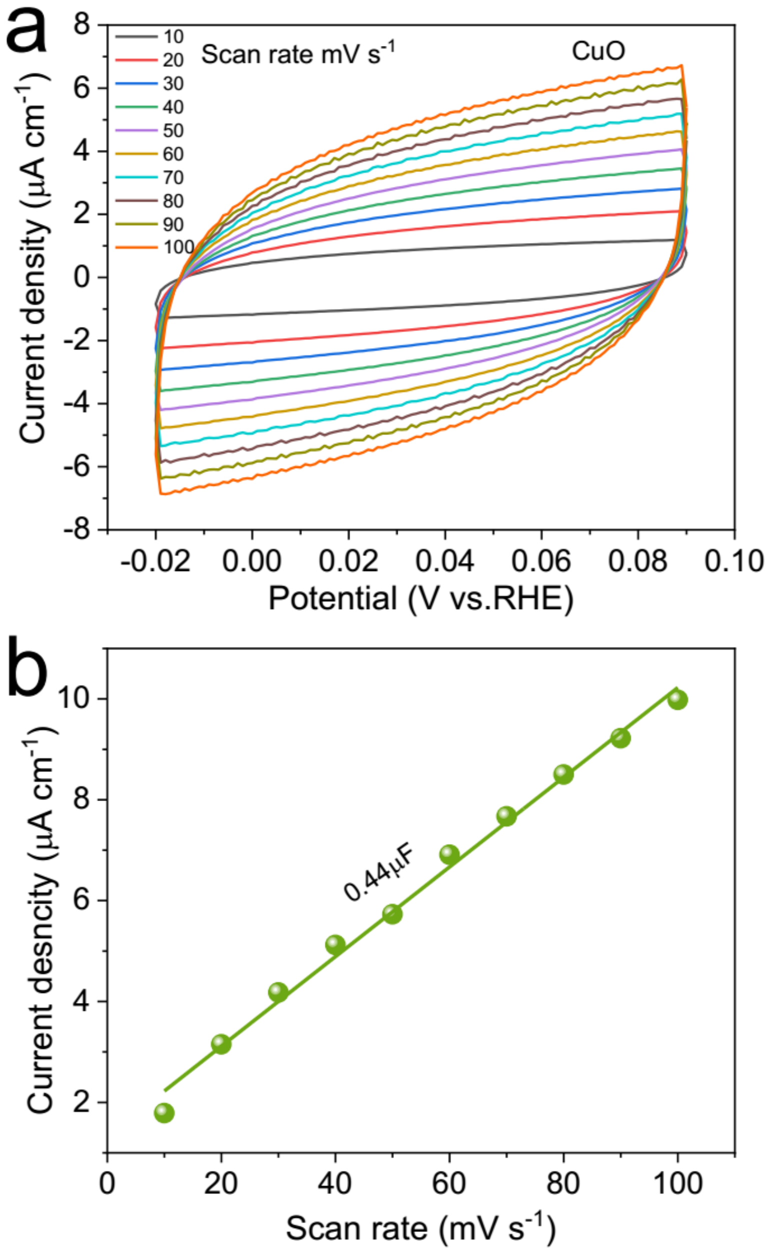

Figure S10. CV curves of CuO at different scan rates (a) and double layer capacitance (b).

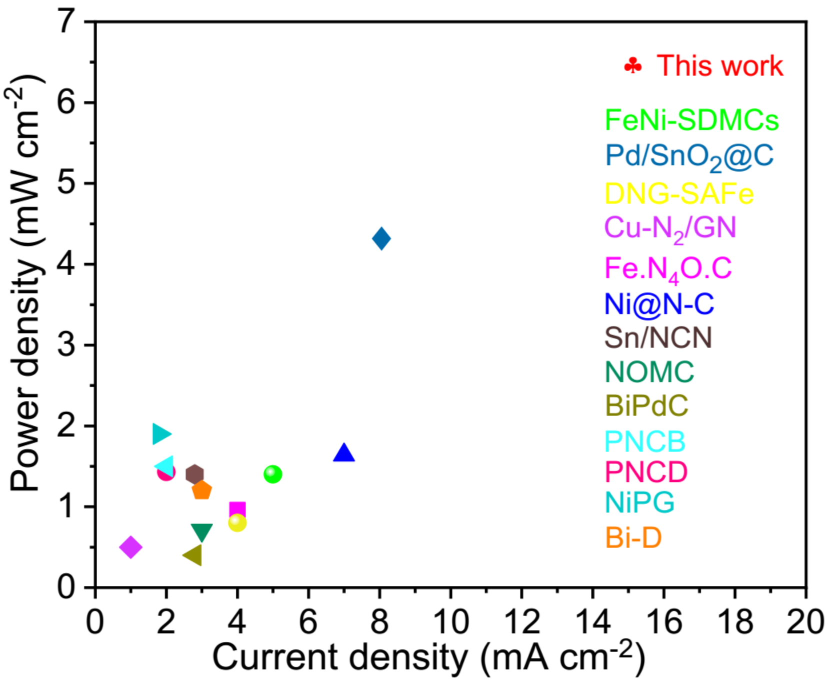

Figure S11. Comparison of the power densities reported in previous studies with that of this work.

Table S1. Comparison of the catalytic CO_2 electroreduction (CO_2ER) performance of $CuO@SnO_2$ with previously reported selective catalysts for ethanol production.

Catalyst	Cell-type	Electrolyte	Potential (V vs. RHE)	j (mA cm^{-2})	FE (%)	Ref.
				CH_3CH_2OH	CH_3CH_2OH	
$CuO@SnO_2$	Flow cell	0.5 M $KHCO_3$	-1.2	-20	41.80%	This work
Cu/Cu_2O	H-cell	0.1 M KCl	-1.1	-32.55	41.20%	[1]
AuCu/Cu-SCA	H-cell	0.5 M $KHCO_3$	-1.0	-5.59	29%	[2]
Cu-NC	H-Cell	0.1 M $KHCO_3$	-1.01	-	18.40%	[3]
$Cu2O/Ag_{2.3\%}$	Flow cell	1M KOH	-2.11	-326.4	40.80%	[4]
CuAg wire	Flow cell	1M KOH	-0.95	-75	25%	[5]
Cu(Ag-20)20	H-cell	0.1 M $KHCO_3$	-1.1	-4.14	14.90%	[6]
$Cu_{1.22}V_{0.19}Se$	Flow cell	1M KOH	-0.8	-207.9	68.30%	[7]

Table S2. Detailed comparison of aqueous Zn-CO_2 battery with previously reported works.

Catalyst	Catholyte	Anolyte	Product	FE (%)	Power density ($mW\ cm^{-2}$)	Energy efficiency (%)	Ref.
**CuO@SnO_2**	**0.8M $KHCO_3$**	**6M KOH + 0.2M $Zn(Ac)_2$**	**Ethanol/Formate**	**36.8/45**	**6.5**	**70.5**	**This work**
Pd/SnO_2@C-MOF	0.8M $KHCO_3$	6M KOH + 0.2M $Zn(Ac)_2$	Formate	95.4	4.29	95.64	[8]
3D porous Pd	1M NaCl + 0.1M NaAc	1M KOH + 0.02M $Zn(Ac)_2$	Formate	81.2	N/A	81.2	[9]
CoPc@DNHCS-8	0.8M $KHCO_3$	0.8M KOH + 0.02M $Zn(Ac)_2$	CO	95.68	1.02	46.7	[10]
Ir@Au	0.8M $KHCO_3$	0.8M KOH + 0.02M $Zn(Ac)_2$	CO	90	N/A	68	[11]
NiFe DASC	2M KCl	2M KOH + 0.02M $Zn(Ac)_2$	CO	90.6	1.35	56.3	[12]
Cu_3P/C	0.1M $KHCO_3$	4M NaOH	CO	47	2.26	N/A	[13]
CB-NGC-2	0.8M $KHCO_3$ + 0.2M$Zn(Ac)_2$	0.8M KOH + 0.2M $Zn(Ac)_2$	CO	91	0.51	N/A	[14]
CNTs@Cu	[EMIM][BF_4]	EMIM][BF_4]	CH_4	93.3	1.89	N/A	[15]
CHF	[EMIM][BF_4]	[EMIM][BF_4]	CH_4	94	0.8	N/A	[16]
Ni–Nx-2D/NPC	1M $KHCO_3$	1M KOH + 0.02M $Zn(Ac)_2$	CO	~100	N/A	53.7	[17]
ZrO2@Ni-NC	0.5M $KHCO_3$	6M KOH + 0.02M $Zn(Ac)_2$	CO	98.6	N/A	76.4	[18]
Bi-D	2M $KHCO_3$ + 0.02M $Zn(Ac)_2$	2M KOH + 0.02M $Zn(Ac)_2$	Formate	93.9	1.16	N/A	[19]
s-SnLi	6M KOH + 0.02M $Zn(Ac)_2$	6M KOH + 0.02M $Zn(Ac)_2$	Formate	92	1.24	N/A	[20]

VO-rich N–SnO_2 NS	0.1M $KHCO_3$	N/A	Formate	83	3.67	56	[21]
CA/N–Ni	0.5M $KHCO_3$	6M KOH + 0.2M $Zn(Ac)_2$	CO	98	0.5	57	[22]
Zn/NC NS	0.5M $KHCO_3$	6M KOH + 0.2M $Zn(Ac)_2$	CO	95	1.8	59	[23]
Ni–N_3-NCNFs	0.5M $KHCO_3$	6M KOH + 0.2M $Zn(Ac)_2$	CO	96.6	1.05	N/A	[24]
NOMC	0.8M $KHCO_3$	6M KOH + 0.2 M $Zn(Ac)_2$	CO	~100	0.71	52.8	[25]
NiPG	3M $KHCO_3$ + 1.5M KCl	6M KOH + 0.2M $Zn(Ac)_2$	CO	66	0.28	61	[26]
PNCB	2M $KHCO_3$ + 0.02M $Zn(Ac)_2$	2M KOH + 0.02M $Zn(Ac)_2$	Formate	94.8	1.43	45	[27]
In/ZnO@C	0.8M $KHCO_3$	0.8M KOH + 0.02M $Zn(Ac)_2$	Formate	90	1.32	61.8	[28]
Cu–N_2/GN	0.1M $KHCO_3$	6M KOH + 0.2M $Zn(Ac)_2$	CO	81	0.6	61	[29]
Ni_9Cu_1@NCNTs/CFM	0.25M $KHCO_3$	N/A	CO	97	0.65	N/A	[30]
Fe-SA/BNC	0.8M $KHCO_3$	0.8M KOH + 0.02 M $Zn(Ac)_2$	CO	94	1.18	63.6	[31]
Fe–P–C	1M $KHCO_3$	6M KOH + 0.02M $Zn(Ac)_2$	CO	95	0.85	75	[32]
Fe_1NC/S_1-1000	0.8M $KHCO_3$	0.8M KOH + 0.02M $Zn(Ac)_2$	CO	96	0.526	N/A	[33]
HPC-Co/CoPc	1M $KHCO_3$	6M KOH + 0.2M $Zn(Ac)_2$	CO+H_2	N/A	2.67	67.5	[34]
Fe–Ni–N–C	0.8M $KHCO_3$	0.8MKOH + 0.02M $Zn(Ac)_2$	CO	93.4	N/A	65	[35]

References

1. Kim, C. Cho, K. M. Park, K. Kim, J. Y. Yun, G. T. Toma, F. M. Gereige, I. and Jung, H. T., "Cu/Cu2O interconnected porous aerogel catalyst for highly productive electrosynthesis of ethanol from CO2" *Advanced Functional Materials*, 2021, 31, 2102142.
2. Shen, S. Peng, X. Song, L. Qiu, Y. Li, C. Zhuo, L. He, J. Ren, J. Liu, X. and Luo, J., "AuCu alloy nanoparticle embedded Cu submicrocone arrays for selective conversion of CO2 to ethanol" *Small*, 2019, 15, 1902229.
3. Cheng, Y.-S. Chu, X.-P. Ling, M. Li, N. Wu, K.-L. Wu, F.-H. Li, H. Yuan, G. and Wei, X.-W., "An MOF-derived copper@ nitrogen-doped carbon composite: The synergistic effects of N-types and copper on selective CO 2 electroreduction" *Catalysis Science & Technology*, 2019, 9, 5668-5675.
4. Wang, P. Yang, H. Tang, C. Wu, Y. Zheng, Y. Cheng, T. Davey, K. Huang, X. and Qiao, S.-Z., "Boosting electrocatalytic CO2–to–ethanol production via asymmetric C–C coupling" *Nature Communications*, 2022, 13, 3754.
5. á Hoang, T. á Verma, S. á Ma, S. á Fister, T. and á Timoshenko, J., "AI áFrenkel, PJA áKenis, AA áGewirth" *J. Am. Chem. Soc*, 2018, 140, 5791.
6. Ting, L. R. L. Pique, O. Lim, S. Y. Tanhaei, M. Calle-Vallejo, F. and Yeo, B. S., "Enhancing CO2 electroreduction to ethanol on copper–silver composites by opening an alternative catalytic pathway" *ACS Catalysis*, 2020, 10, 4059-4069.
7. Sun, W. Wang, P. Jiang, Y. Jiang, Z. Long, R. Chen, Z. Song, P. Sheng, T. Wu, Z. and Xiong, Y., "V-doped Cu2Se hierarchical nanotubes enabling flow-cell CO2 electroreduction to ethanol with high efficiency and selectivity" *Advanced Materials*, 2022, 34, 2207691.
8. Aslam, M. K. Wang, H. Nie, Z. Chen, S. Li, Q. and Duan, J., "Unlock flow-type reversible aqueous Zn-CO(2) batteries" *Mater Horiz*, 2024, 11, 2657-2666.
9. Xie, J. Wang, X. Lv, J. Huang, Y. Wu, M. Wang, Y. and Yao, J., "Reversible aqueous Zinc–CO2 batteries based on CO2–HCOOH interconversion" *Angewandte Chemie International Edition*, 2018, 57, 16996-17001.
10. Gong, S. Wang, W. Zhang, C. Zhu, M. Lu, R. Ye, J. Yang, H. Wu, C. Liu, J. Rao, D. Shao, S. and Lv, X., "Tuning the Metal Electronic Structure of Anchored Cobalt Phthalocyanine via Dual-Regulator for Efficient CO2 Electroreduction and Zn–CO2 Batteries" *Advanced Functional Materials*, 2022, 32, 2110649.

11. Wang, X. Xie, J. Ghausi, M. A. Lv, J. Huang, Y. Wu, M. Wang, Y. and Yao, J., "Rechargeable Zn–CO2 Electrochemical Cells Mimicking Two-Step Photosynthesis" *Advanced Materials*, 2019, 31, 1807807.
12. Zeng, Z. Gan, L. Y. Bin Yang, H. Su, X. Gao, J. Liu, W. Matsumoto, H. Gong, J. Zhang, J. Cai, W. Zhang, Z. Yan, Y. Liu, B. and Chen, P., "Orbital coupling of hetero-diatomic nickel-iron site for bifunctional electrocatalysis of CO2 reduction and oxygen evolution" *Nature Communications*, 2021, 12, 4088.
13. Peng, M. Y. Ci, S. Q. Shao, P. Cai, P. W. and Wen, Z. H., "Cu3P/C Nanocomposites for Efficient Electrocatalytic CO2 Reduction and Zn-CO2 Battery" *Journal of nanoscience and nanotechnology*, 2019, 19, 3232-3236.
14. Hao, X. An, X. Patil, A. M. Wang, P. Ma, X. Du, X. Hao, X. Abudula, A. and Guan, G., "Biomass-Derived N-Doped Carbon for Efficient Electrocatalytic CO2 Reduction to CO and Zn–CO2 Batteries" *Acs Applied Materials & Interfaces*, 2021, 13, 3738-3747.
15. Chen, Y. Mei, Y. Li, M. Dang, C. Huang, L. Wu, W. Wu, Y. Yu, X. Wang, K. Gu, L. Liu, L. and Cao, X., "Highly selective CO2 conversion to methane or syngas tuned by CNTs@non-noble–metal cathodes in Zn–CO2 flow batteries" *Green Chemistry*, 2021, 23, 8138-8146.
16. Wang, K. Wu, Y. Cao, X. Gu, L. and Hu, J., "A Zn–CO2 Flow Battery Generating Electricity and Methane" *Advanced Functional Materials*, 2020, 30, 1908965.
17. Zeng, Z. Mohamed, A. G. A. Zhang, X. and Wang, Y., "Wide Potential CO2-to-CO Electroreduction Relies on Pyridinic-N/Ni–Nx Sites and Its Zn–CO2 Battery Application" *Energy Technology*, 2021, 9, 2100205.
18. Wang, X. Feng, S. Lu, W. Zhao, Y. Zheng, S. Zheng, W. Sang, X. Zheng, L. Xie, Y. Li, Z. Yang, B. Lei, L. Wang, S. and Hou, Y., "A New Strategy for Accelerating Dynamic Proton Transfer of Electrochemical CO2 Reduction at High Current Densities" *Advanced Functional Materials*, 2021, 31, 2104243.
19. Wang, Y. Huang, Z. Lei, Y. Wu, J. Bai, Y. Zhao, X. Liu, M. Zhan, L. Tang, S. Zhang, X. Luo, F. and Xiong, X., "Bismuth with abundant defects for electrocatalytic CO2 reduction and Zn–CO2 batteries" *Chemical Communications*, 2022, 58, 3621-3624.
20. Yan, S. Peng, C. Yang, C. Chen, Y. Zhang, J. Guan, A. Lv, X. Wang, H. Wang, Z. Sham, T.-K. Han, Q. and Zheng, G., "Electron Localization and Lattice Strain Induced by Surface Lithium Doping Enable Ampere-Level Electrosynthesis of

Formate from CO2" *Angewandte Chemie International Edition*, 2021, 60, 25741-25745.

21. Li, Z. Cao, A. Zheng, Q. Fu, Y. Wang, T. Arul, K. T. Chen, J.-L. Yang, B. Adli, N. M. Lei, L. Dong, C.-L. Xiao, J. Wu, G. and Hou, Y., "Elucidation of the Synergistic Effect of Dopants and Vacancies on Promoted Selectivity for CO2 Electroreduction to Formate" *Advanced Materials*, 2021, 33, 2005113.
22. Zhang, Y. Wang, X. Zheng, S. Yang, B. Li, Z. Lu, J. Zhang, Q. Adli, N. M. Lei, L. Wu, G. and Hou, Y., "Hierarchical Cross-Linked Carbon Aerogels with Transition Metal-Nitrogen Sites for Highly Efficient Industrial-Level CO2 Electroreduction" *Advanced Functional Materials*, 2021, 31, 2104377.
23. Chen, J. Li, Z. Wang, X. Sang, X. Zheng, S. Liu, S. Yang, B. Zhang, Q. Lei, L. Dai, L. and Hou, Y., "Promoting CO2 Electroreduction Kinetics on Atomically Dispersed Monovalent ZnI Sites by Rationally Engineering Proton-Feeding Centers" *Angewandte Chemie International Edition*, 2022, 61, e202111683.
24. Zheng, W. Wang, Y. Shuai, L. Wang, X. He, F. Lei, C. Li, Z. Yang, B. Lei, L. Yuan, C. Qiu, M. Hou, Y. and Feng, X., "Highly Boosted Reaction Kinetics in Carbon Dioxide Electroreduction by Surface-Introduced Electronegative Dopants" *Advanced Functional Materials*, 2021, 31, 2008146.
25. Gao, S. Liu, Y. Xie, Z. Qiu, Y. Zhuo, L. Qin, Y. Ren, J. Zhang, S. Hu, G. Luo, J. and Liu, X., "Metal-Free Bifunctional Ordered Mesoporous Carbon for Reversible Zn-CO2 Batteries" *Small Methods*, 2021, 5, 2001039.
26. Yang, R. Xie, J. Liu, Q. Huang, Y. Lv, J. Ghausi, M. A. Wang, X. Peng, Z. Wu, M. and Wang, Y., "A trifunctional Ni–N/P–O-codoped graphene electrocatalyst enables dual-model rechargeable Zn–CO 2/Zn–O 2 batteries" *Journal of Materials Chemistry A*, 2019, 7, 2575-2580.
27. Wang, Y. Xu, L. Zhan, L. Yang, P. Tang, S. Liu, M. Zhao, X. Xiong, Y. Chen, Z. and Lei, Y., "Electron accumulation enables Bi efficient CO2 reduction for formate production to boost clean Zn-CO2 batteries" *Nano Energy*, 2022, 92, 106780.
28. Teng, X. Niu, Y. Gong, S. Xu, M. Liu, X. Ji, L. and Chen, Z., "In/ZnO@C hollow nanocubes for efficient electrochemical reduction of CO2 to formate and rechargeable Zn–CO2 batteries" *Materials Chemistry Frontiers*, 2021, 5, 6618-6627.
29. Zheng, W. Yang, J. Chen, H. Hou, Y. Wang, Q. Gu, M. He, F. Xia, Y. Xia, Z. Li, Z. Yang, B. Lei, L. Yuan, C. He, Q. Qiu, M. and Feng, X., "Atomically Defined

Undercoordinated Active Sites for Highly Efficient CO2 Electroreduction" *Advanced Functional Materials*, 2020, 30, 1907658.

30. Shen, S. Han, C. Wang, B. and Wang, Y., "Engineering d-band center of nickel in nickel@nitrogen-doped carbon nanotubes array for electrochemical reduction of CO2 to CO and Zn-CO2 batteries" *Chinese Chemical Letters*, 2022, 33, 3721-3725.

31. Liu, S. Jin, M. Sun, J. Qin, Y. Gao, S. Chen, Y. Zhang, S. Luo, J. and Liu, X., "Coordination environment engineering to boost electrocatalytic CO2 reduction performance by introducing boron into single-Fe-atomic catalyst" *Chemical Engineering Journal*, 2022, 437, 135294.

32. Liu, S. Wang, L. Yang, H. Gao, S. Liu, Y. Zhang, S. Chen, Y. Liu, X. and Luo, J., "Nitrogen-Doped Carbon Polyhedrons Confined Fe–P Nanocrystals as High-Efficiency Bifunctional Catalysts for Aqueous Zn−CO2 Batteries" *Small*, 2022, 18, 2104965.

33. Wang, T. Sang, X. Zheng, W. Yang, B. Yao, S. Lei, C. Li, Z. He, Q. Lu, J. and Lei, L., "Gas Diffusion Strategy for Inserting Atomic Iron Sites into Graphitized Carbon Supports for Unusually High-Efficient CO2 Electroreduction and High-Performance Zn–CO2 Batteries" *Advanced Materials*, 2020, 32, 2002430.

34. Ni, W. Liu, Z. Guo, X. Zhang, Y. Ma, C. Deng, Y. and Zhang, S., "Dual single-cobalt atom-based carbon electrocatalysts for efficient CO2-to-syngas conversion with industrial current densities" *Applied Catalysis B: Environmental*, 2021, 291, 120092.

35. Jiao, L. Zhu, J. Zhang, Y. Yang, W. Zhou, S. Li, A. Xie, C. Zheng, X. Zhou, W. Yu, S.-H. and Jiang, H.-L., "Non-Bonding Interaction of Neighboring Fe and Ni Single-Atom Pairs on MOF-Derived N-Doped Carbon for Enhanced CO2 Electroreduction" *Journal of the American Chemical Society*, 2021, 143, 19417-19424.
